# Preliminary Exploration of Quantitative Stress Myocardial Contrast Echocardiography for Risk Stratification in Hypertrophic Cardiomyopathy: A 5-Year Prospective Study

**DOI:** 10.31083/RCM49216

**Published:** 2026-07-23

**Authors:** Ye Su, Chunmei Li, Lixue Yin

**Affiliations:** ^1^Department of Cardiovascular Ultrasound & Noninvasive Cardiology, Sichuan Provincial People’s Hospital, University of Electronic Science and Technology of China, 610031 Chengdu, Sichuan, China; ^2^Ultrasound in Cardiac Electrophysiology and Biomechanics Key Laboratory of Sichuan Province, 610031 Chengdu, Sichuan, China

**Keywords:** hypertrophic cardiomyopathy, myocardial contrast echocardiography, risk stratification

## Abstract

**Background:**

Hypertrophic cardiomyopathy (HCM) is an autosomal dominant genetic cardiovascular disease caused by mutations in genes encoding sarcomeric proteins. Positron emission tomography (PET) has demonstrated prognostic value in assessing myocardial perfusion; however, the clinical utility of PET is limited by radiation exposure and cost. Myocardial contrast echocardiography (MCE) is a radiation-free, widely available imaging modality that shows promise for assessing microcirculatory function; however, the ability of MCE to predict long-term cardiovascular events in HCM remains unclear.

**Methods:**

This study recruited 142 patients with HCM and 80 healthy controls from the Sichuan Provincial People’s Hospital. All participants underwent exercise-stress MCE to quantify myocardial perfusion using wash-in slope (WIS) and perfusion intensity (PI). Obstructive coronary artery disease was excluded by coronary computed tomography or invasive angiography. A 5-year follow-up was conducted for patients with HCM to monitor adverse cardiovascular events. Subgroup analyses were performed according to clinical event status, left ventricular outflow tract pressure gradient (LVOT-PG), and genetic classification.

**Results:**

The event-positive group showed significantly reduced resting and peak WIS and peak PI, along with complete loss of both WIS and PI reserves. Compared with the variant of uncertain significance (VUS) group, the genetic risk group had significantly lower resting and peak WIS and PI, with a significantly lower WIS reserve (ΔWIS) but comparable PI reserve (ΔPI). Among the resting obstruction, latent obstruction, and non-obstruction subgroups, WIS differed significantly, with the resting obstruction subgroup exhibiting the lowest WIS values and complete loss of both WIS and PI reserves. Resting and peak WIS were strongly and inversely correlated with adverse events. Receiver operating characteristic (ROC) analysis demonstrated that both resting and peak WIS had favorable predictive performance for adverse events.

**Conclusions:**

This preliminary, single-center, hypothesis-generating study suggests that WIS derived from exercise-stress MCE may be a useful marker for risk stratification in HCM; however, larger, multicenter studies are needed to confirm these findings.

## 1. Introduction

The incidence rate of hypertrophic cardiomyopathy (HCM) is as high as 1 in 200 individuals. The primary pathological changes associated with HCM include myocardial cell hypertrophy and proliferation, disordered cellular arrangement, and interstitial fibrosis. The pathophysiological mechanisms involved are highly heterogeneous and contribute significantly to sudden cardiac death (SCD) in young people [1,2]. Coronary microvascular dysfunction (CMD) is a hallmark of HCM pathophysiology and contributes to myocardial ischemia, fibrosis progression, and arrhythmogenesis [3]. Reduced myocardial flow reserve (MFR) measured by PET is an independent predictor of clinical deterioration and death in patients with HCM, indicating that CMD is closely associated with adverse outcomes [4]. However, the limited availability of PET and its associated radiation exposure restricts its routine clinical use. In contrast, myocardial contrast echocardiography (MCE) offers a bedside-capable, ionizing radiation-free alternative for quantifying microvascular perfusionby tracking the replenishment kinetics of intravenously infused microbubbles. Soliman et al. [5] reported that in HCM, impaired myocardial perfusion reserve arose from both reduced myocardial blood volume reserve and elevated diastolic pressure, and correlated independently with left ventricular outflow tract gradient, end-diastolic pressure, and mass index. These observations support the use of quantitative MCE for non-invasive assessment of CMD and its potential role in risk stratification. MCE may er greater sensitivity than dobutamine stress echocardiography for detecting ischemia in HCM, as perfusion abnormalities are thought to be primarily driven by coronary microvascular dysfunction and rarefaction rather than by epicardial coronary artery disease [6]. These findings align with histopathological studies linking capillary density reduction to replacement fibrosis [7], suggesting MCE provides a functional counterpart to CMR’s late gadolinium enhancement (LGE). MCE has been validated against ^13^N-ammonia PET for the assessment of absolute myocardial blood flow, which demonstrated excellent correlation over a wide range of rest and hyperemic flows (r^2^ = 0.92) and close agreement for corresponding myocardial flow reserve (r^2^ = 0.81) [8]. Therefore, this study tests the hypothesis that MCE-derived myocardial perfusion parameters, particularly the wash-in slope (WIS), can predict major adverse cardiovascular events in patients with HCM.

## 2. Materials and Methods

### 2.1 Subjects

Inclusion criteria for HCM: The diagnosis of HCM was in accordance with the “Guidelines for the Diagnosis and Management of Hypertrophic Cardiomyopathy” published by the ESC in 2014 [9], and the patient had a genetic test report. The exclusion criteria: coronary heart disease, ischemic cardiomyopathy, congestive heart failure, hypertension, and other diseases that may cause myocardial hypertrophy. After excluding patients with poor image quality and those who could not participate in the 5-year follow-up, a total of 142 HCM patients and 80 healthy controls were included in the study. All participants provided informed consent, and the study was approved by the Ethics Committee of Sichuan Provincial People’s Hospital.

A 5-year follow-up was conducted on the HCM patients included in the study. These patients were monitored monthly and categorized into two groups based on their clinical outcomes: an event-positive group, comprising those with unexplained syncope, ventricular tachycardia, defibrillator implantation, atrial fibrillation [10,11,12,13], or heart failure, and the non-event group. Additionally, patients were classified into obstruction or non-obstruction groups based on their LVOT-PG <30 or ≥30 mmHg [9]. They were also divided into the genetic risk or variant of uncertain significance (VUS) groups based on genetic test results.

### 2.2 Equipment and Materials

All the HCM patients included in the study had genetic test reports, and their evaluations included resting MCE, exercise electrocardiography, and peak MCE. HCM patients did not take beta-blockers before the exercise test and were not informed of any medication changes.

For exercise testing, the SunTech Tango motion blood pressure monitor (SunTech Medical Instruments Company, Morrisville, North Carolina, USA) and the Mortara XScribe motion running analyzer (Mortara Instruments Company, Milwaukee, Wisconsin, USA) were used. The testing was performed according to the BRUCE protocol and adhered to the 2002 American College of Cardiology/American Heart Association exercise testing guidelines [14].

The MCE test utilized the Philips EPIQ7C ultrasound system (Philips Healthcare, Andover, MA, USA) and X5-1 transducer (1.0–5.0 MHz; Philips Healthcare, Andover, MA, USA), QLAB13 quantitative analysis software (Philips Healthcare, Andover, MA, USA), and the second-generation ultrasound contrast agent “SonoVue” (Bracco, Società per Azioni, Milan, Italy). Resting stage: A 10 mL syringe was prepared by drawing 2 mL of SonoVue suspension and 8 mL of 0.9% NaCl (normal saline). The Philips EPIQ7C ultrasound system was set to “MCE mode”, and the contrast agent was administered via the median cubital vein over approximately 2 minutes. Following the injection, 3 mL of normal saline was administered at the same rate. The X5-1 transducer was employed to acquire dynamic images in the apical four-chamber (A4C), three-chamber (A3C), and two-chamber (A2C) views. The cardiac cycle was continuously recorded for at least 15 cycles. All dynamic images were acquired intermittently in “FLASH” mode to induce microbubble contrast in myocardial tissue. Peak stage: dynamic images from the A4C, A2C, and A3C views were recorded for at least 15 cardiac cycles, and were required to be consistent with those obtained at rest.

### 2.3 Analysis

**Fig. 1.**
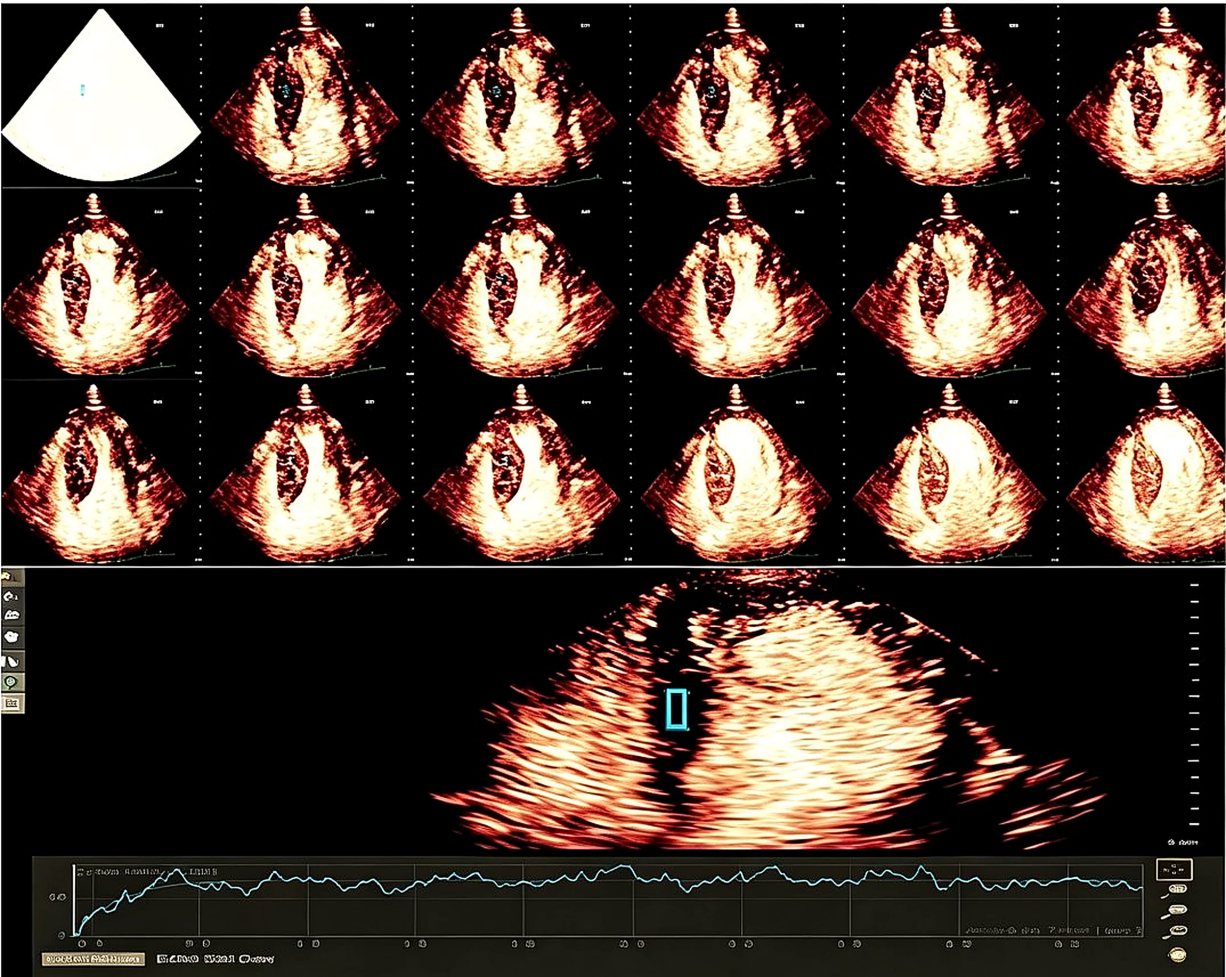
**Myocardial perfusion analysis in HCM**. HCM, hypertrophic cardiomyopathy.

All operations and analyses were performed in accordance with the guidelines of the American Society of Echocardiography (ASE) [15]. General clinical data: gender, age, body surface area (BSA), body mass index (BMI), left ventricular mass index (LVMI), maximum thickness of the chamber wall (maxRW), the rest and peak perfusion wash-in slope (R_WIS, P_WIS), the rest and peak perfusion time to peak (R_TTP, P_TTP), the rest and peak perfusion intensity (R_PI, P_PI), the rest and peak perfusion rise time (R_RT, P_RT), the resting number of sparse myocardial segments for perfusion (R_Sparse_num), the peak number of sparse myocardial segments for perfusion (P_Sparse_num), peak minus rest (Δ), as shown in Fig. 1.

### 2.4 Statistical Methods

Quantitative data are expressed as mean ± standard deviation (SD), while categorical data are expressed as percentages. The normality of continuous variables was assessed using the Shapiro–Wilk test. Comparisons between two groups were performed using the independent-samples *t*-test for normally distributed data or the Mann–Whitney U test for non-normally distributed data. Categorical variables were analyzed using either the chi-square test or Fisher’s test. Correlation analyses were performed using Pearson’s correlation coefficient (r) for normally distributed variables and Spearman’s rank correlation coefficient (rₛ) for non‑normally distributed variables. To evaluate the predictive performance of the WIS for the binary endpoint (event occurrence vs. non‑occurrence) over the 5‑year follow‑up period, receiver operating characteristic (ROC) curves were generated and the area under the curve (AUC) was calculated. Given the limited number of endpoint events (n = 40) and the exploratory, hypothesis‑generating nature of this study, multivariable adjustment was not performed to avoid model overfitting. The primary analysis therefore focused on the univariate predictive performance of WIS as quantified by the ROC. The optimal threshold for WIS was determined using the Youden index, with corresponding sensitivity and specificity reported. The 95% confidence interval for the AUC was calculated using the DeLong method. A two-tailed *p*-value < 0.05 was considered statistically significant. Statistical analyses were conducted using SPSS 23.0 (IBM Corp., Armonk, NY, USA), R 4.2.0 (R Foundation for Statistical Computing, Vienna, Austria), and Stata 17.0 (StataCorp LLC, College Station, TX, USA).

## 3. Results

### 3.1 Follow-Up of Positive Events and Grouping

According to the study’s follow-up plan, the clinical endpoint was defined as the first occurrence of any component of the composite endpoint. Among the 142 HCM patients, 40 (28.17%) experienced at least one adverse event during the 5-year follow-up period. The distribution of individual events is presented in Table 1A: there were 6 cases of unexplained syncope, 15 cases of heart failure, 6 cases requiring implantable cardioverter-defibrillator (ICD) implantation, 4 cases of ventricular tachycardia, and 9 cases of atrial fibrillation. Table 1B shows the corresponding percentages, calculated based on the total number of patients in the positive event group (n = 40). Each patient experienced only one type of event during follow-up.

**Table 1A.** **Positive events in HCM**.

	Unexplained syncope	Heart failure	ICD implantation	Ventricular tachycardia	Atrial fibrillation
Positive events (n = 40)	6 cases	15 cases	6 cases	4 cases	9 cases

n, sample size; ICD, implantable cardioverter-defibrillator.

**Table 1B.** **Distribution of individual endpoint components in the positive event group (n = 40)**.

Endpoint component	n	%
Unexplained syncope	6 cases	15%
Heart failure	15 cases	37.5%
ICD implantation	6 cases	15%
Ventricular tachycardia	4 cases	10%
Atrial fibrillation	9 cases	22.5%
Total	40	100

n, sample size; ICD, implantable cardioverter-defibrillator.

### 3.2 Genetic Testing

As indicated in the genetic test reports for the HCM patients (Table 2), 21 genes were classified as VUS, while 16 were identified as pathogenic. Among these, 8 genes overlapped: *MYH7* (myosin heavy chain 7), *MYBPC3* (myosin binding protein C3), *TNNI2* (troponin I2, fast skeletal type), *ACTN2* (actinin alpha 2), *MYPN* (myopalladin), *LDB3* (LIM domain binding 3), *RYR2* (ryanodine receptor 2), and *MYLK2* (myosin light chain kinase 2). According to American College of Medical Genetics and Genomics (ACMG) standards [16], patients with pathogenic or likely pathogenic findings from genetic testing were categorized into the genetic risk group, while those with VUS were classified into the VUS group.

**Table 2.** **Genetic testing results**.

VUS	MYH7	MYBPC3	TNNI2	LDB3	RYR2	MYPN	TNN
Sample (n)	25	1	2	3	5	1	19
VUS	ACTN2	MYLK2	SCN5A	PRDM16	CTNNA3	FLNC	KCNH2
Sample (n)	1	2	1	1	2	2	1
VUS	DMD	DYSF	BAG3	PSEN2	RBM20	ALPK3	TMPO
Sample (n)	3	1	1	1	1	1	1
Genetic risk	MYH7	MYBPC3	TNNI2	TNNI3	LDB3	RYR2	MYPN
Sample (n)	16	24	5	4	3	1	1
Genetic risk	ACTN2	MYLK2	MYOZ2	TPM1	LAMP2	FHL1	SYNE2
Sample (n)	1	1	1	1	1	1	1
Genetic risk	DES	DSP					
Sample (n)	1	5					

n, the number of genotypes. The same gene may appear in both the VUS and genetic risk groups, as different variants within the same gene can have different ACMG classifications. VUS, variant of uncertain significance; ACTN2, actinin alpha 2; BAG3, BCL2 associated athanogene 3; DES, desmin; DMD, dystrophin; DSP, desmoplakin; DYSF, dysferlin; LDB3, LIM domain binding 3; MYBPC3, myosin binding protein C3; MYH7, myosin heavy chain 7; MYLK2, myosin light chain kinase 2; MYOZ2, myozenin 2; MYPN, myopalladin; PRDM16, PR/SET domain 16; PSEN2, presenilin 2; RYR2, ryanodine receptor 2; SCN5A, sodium voltage-gated channel alpha subunit 5; TNNI2, troponin I2, fast skeletal type; TNNI3, troponin I3, cardiac type; TPM1, tropomyosin 1.

### 3.3 General and Myocardial Perfusion Between the HCM and Control Groups

All participants underwent stress MCE without experiencing any allergic reactions. In the HCM group, the resting and peak microcirculation perfusion parameters—WIS, TTP, RT, and PI—were all significantly lower (see Table 3).

**Table 3.** **General and myocardial perfusion between the HCM group and normal group**.

	HCM group	Normal group	*p*
Sample	142	80	NA
Male, n (%)	98 (69.01)	56 (70.00)	0.878
Age, years (mean (SD))	49.09 (14.17)	47.98 (10.61)	0.542
BSA (mean (SD)) (m^2^)	1.73 (0.20)	1.73 (0.20)	0.977
BMI (mean (SD)) (kg/m^2^)	24.10 (3.54)	24.03 (3.29)	0.882
LVMI (mean (SD)) (g/m^2^)	145.89 (60.56)	109.22 (26.13)	<0.001*
R_HR (mean (SD)) (bpm)	77.56 (12.27)	88.17 (11.22)	<0.001*
maxRW (mean (SD)) (mm)	20.48 (4.75)	9.10 (1.12)	<0.001*
R_WIS (mean (SD)) (m/s)	5.66 (3.87)	14.20 (3.49)	<0.001*
R_TTP (mean (SD)) (s)	5.11 (1.89)	1.83 (0.59)	<0.001*
R_PI (mean (SD)) (dB)	8.30 (3.44)	12.68 (3.01)	<0.001*
R_RT (mean (SD)) (s)	2.69 (1.79)	0.74 (0.63)	<0.001*
P_HR (mean (SD)) (bpm)	155.40 (25.31)	159.18 (13.30)	0.147
P_WIS (mean (SD)) (m/s)	8.23 (6.34)	32.86 (11.44)	<0.001*
P_TTP (mean (SD)) (s)	2.93 (1.84)	0.78 (0.48)	<0.001*
P_PI (mean (SD)) (dB)	9.41 (4.13)	17.71 (3.79)	<0.001*
P_RT (mean (SD)) (s)	1.39 (1.24)	0.50 (0.61)	<0.001*

*p < 0.05. BSA, body surface area; BMI, body mass index; LVMI, left ventricular mass index; HR, heart rate; maxRW, maximum thickness of the chamber wall; Risk gene, pathogenic gene; R_WIS, the rest perfusion wash-in slope; P_WIS, the peak perfusion wash-in slope; R_TTP, rest perfusion time to peak; P_TTP, peak perfusion time to peak; R_PI, rest perfusion intensity; P_PI, peak perfusion intensity; R_RT, the rest and peak perfusion rise time; P_RT, peak perfusion rise time; NA, not applicable.

### 3.4 General Data and Myocardial Perfusion Between the Genetic Risk Group and the VUS Group

**Fig. 2.**
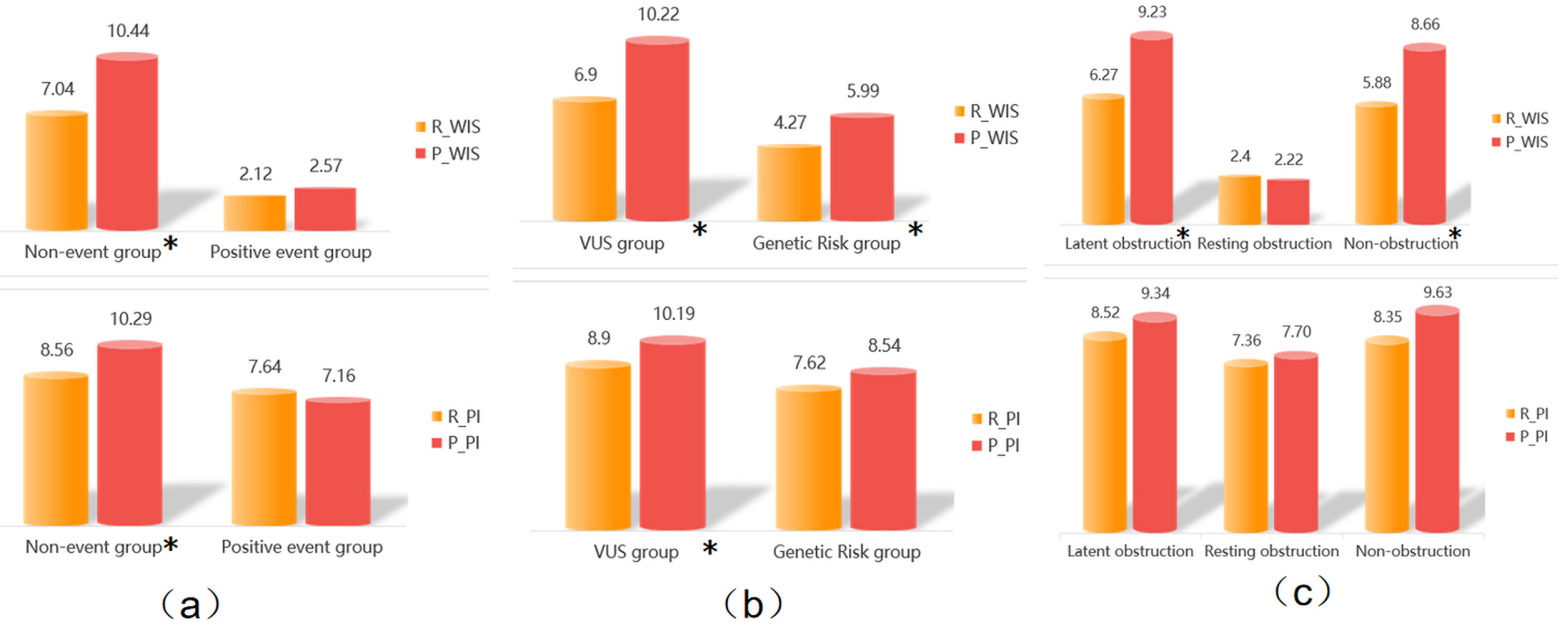
**Comparison of resting and peak WIS and PI**. Within-group comparisons of WIS and PI at rest and peak, stratified by subgroups. (a) Positive event vs. non-event groups. (b) Variants of uncertain significance (VUS) vs. genetic risk groups. (c) Resting obstruction, latent obstruction, and non-obstruction groups. R_WIS, the rest perfusion wash-in slope; P_WIS, the peak perfusion wash-in slope; R_PI, rest perfusion intensity; P_PI, peak perfusion intensity. *p < 0.05.

As shown in Table 4, the genetic risk group exhibited worse resting and peak WIS, PI, and reserve function, with no significant difference in left ventricular mass index (LVMI) between the two groups. Notably, there was a significant difference in WIS before and after exercise in both the VUS and Genetic Risk groups (Fig. 2b).

**Table 4.** **Myocardial perfusion data between the risk gene group and the VUS group**.

	VUS group	Genetic risk group	*p*
Sample	75	67	
LVMI (mean (SD)) (g/m^2^)	148.87 (71.44)	142.55 (45.73)	0.527
Male, n (%)	59 (78.67)	39 (58.20)	0.014*
Age, years (mean (SD))	46.96 (12.28)	51.48 (15.77)	0.058
BSA (mean (SD)) (m^2^)	1.77 (0.18)	1.69 (0.23)	0.033*
BMI (mean (SD)) (kg/m^2^)	24.19 (3.32)	23.90 (3.78)	0.631
Risk gene, n (%)	0 (0.00)	67 (100.00)	<0.001*
Positive event, n (%)	4 (5.33)	36 (53.73)	<0.001*
Obstruction, n (%)	14 (18.67)	25 (37.31)	0.022*
R_WIS (mean (SD)) (m/s)	6.90 (3.32)	4.27 (4.00)	<0.001*
R_TTP (mean (SD)) (s)	4.99 (1.81)	5.26 (1.97)	0.395
R_PI (mean (SD)) (dB)	8.90 (3.26)	7.62 (3.53)	0.026*
R_RT (mean (SD)) (s)	2.65 (1.74)	2.74 (1.86)	0.762
R_Sparse_num (mean (SD)) (number)	5.35 (4.23)	8.85 (5.78)	<0.001*
P_WIS (mean (SD)) (m/s)	10.22 (6.10)	5.99 (5.87)	<0.001*
P_TTP (mean (SD)) (s)	2.38 (1.55)	3.53 (1.96)	<0.001*
P_PI (mean (SD)) (dB)	10.19 (4.47)	8.54 (3.56)	0.017*
P_RT (mean (SD)) (s)	1.03 (0.88)	1.80 (1.45)	<0.001*
P_Sparse_num (mean (SD)) (number)	4.75 (4.39)	8.70 (5.68)	<0.001*
ΔTTP (mean (SD)) (s)	–2.60 (1.60)	–1.72 (2.09)	0.005*
ΔPI (mean (SD)) (dB)	1.28 (4.87)	0.92 (4.40)	0.644
ΔRT (mean (SD)) (s)	–1.61 (1.75)	–0.94 (2.15)	0.042*
ΔWIS (mean (SD)) (m/s)	3.32 (4.74)	1.72 (3.74)	0.026*

*p < 0.05. BSA, Body surface area; BMI, Body Mass Index; LVMI, left ventricular mass index; HR, heart rate; maxRW, Maximum thickness of the chamber wall; Risk gene, pathogenic gene; R_WIS, the rest perfusion wash-in slope; P_WIS, the peak perfusion wash-in slope; R_TTP, rest perfusion time to peak; P_TTP, peak perfusion time to peak; R_PI, rest perfusion intensity; P_PI, peak perfusion intensity; R_RT, the rest and peak perfusion rise time; P_RT, peak perfusion rise time.; Δ, peak minus rest; R_ Sparse_num, the resting number of sparse myocardial segments for perfusion; P_Sparse_num, the peak number of sparse myocardial segments for perfusion.

### 3.5 General Data and Myocardial Perfusion Between the Non-Obstruction, Resting Obstruction and Latent Obstruction Groups

Table 5 indicates there were 12 cases of resting obstruction and 27 cases of exercise-induced obstruction among HCM patients. In this study, both resting obstruction and latent obstruction were included in the obstruction group. Although there were no significant differences in LVMI, the three groups showed statistically significant differences in resting and peak WIS, P_TTP, and P_RT. In the Latent Obstruction group and the non-obstruction group, there were significant differences in WIS before and after exercise. However, the Resting Obstruction group showed no difference in WIS before and after exercise (Fig. 2c).

**Table 5.** **Myocardial perfusion between the Non/resting/latent obstruction group**.

	Latent obstruction	Resting obstruction	Non-obstruction	*p*
Sample	27	12	103	NA
LVMI (mean (SD)) (g/m^2^)	129.96 (50.43)	141.35 (43.37)	150.60 (64.24)	0.280
R_WIS (mean (SD)) (m/s)	6.27 (3.63)	2.40 (1.91)	5.88 (3.94)	0.008*
R_TTP (mean (SD)) (s)	4.68 (1.85)	5.89 (1.67)	5.14 (1.91)	0.178
R_PI (mean (SD)) (dB)	8.52 (3.23)	7.36 (4.82)	8.35 (3.32)	0.601
R_RT (mean (SD)) (s)	2.44 (1.56)	2.41 (1.47)	2.79 (1.88)	0.573
P_WIS (mean (SD)) (m/s)	9.23 (6.59)	2.22 (0.79)	8.66 (6.30)	0.002*
P_TTP (mean (SD)) (s)	2.92 (2.01)	4.73 (1.50)	2.72 (1.72)	0.001*
P_PI (mean (SD)) (dB)	9.34 (3.95)	7.70 (2.94)	9.63 (4.28)	0.311
P_RT (mean (SD)) (s)	1.54 (1.69)	2.29 (1.15)	1.25 (1.06)	0.018*
ΔTTP (mean (SD)) (s)	–1.77 (2.27)	–1.16 (2.04)	–2.42 (1.72)	0.039*
ΔPI (mean (SD)) (dB)	0.82 (4.57)	0.33 (5.84)	1.27 (4.55)	0.756
ΔRT (mean (SD)) (s)	–0.90 (2.19)	–0.12 (2.00)	–1.53 (1.86)	0.032*
ΔWIS (mean (SD)) (m/s)	2.96 (4.68)	–0.17 (1.92)	2.78 (4.39)	0.073

*p < 0.05. BSA, body surface area; BMI, body mass index; LVMI, left ventricular mass index; HR, heart rate; maxRW, maximum thickness of the chamber wall; LVOT-PG, left ventricular outflow tract pressure gradient; Risk gene, pathogenic gene; R_WIS, the rest perfusion wash-in slope; P_WIS, the peak perfusion wash-in slope; R_TTP, rest perfusion time to peak; P_TTP, peak perfusion time to peak; R_PI, rest perfusion intensity; P_PI, peak perfusion intensity; R_RT, the rest and peak perfusion rise time; P_RT, peak perfusion rise time; Δ, peak minus rest; NA, not applicable.

### 3.6 General Data and Myocardial Perfusion Between the Positive Events Group and the Non-Event Group

No significant differences in LVMI were observed between the positive event group and the non-event group. However, the resting and peak WIS values in the positive event group were significantly lower (Table 6). In both the latent obstruction and the non-obstruction groups, significant differences in WIS were noted before and after exercise. There was a significant difference in WIS before and after the exercise in the non-event group, whereas no such difference was observed in the Positive event group (Fig. 2a).

**Table 6.** **Myocardial perfusion data between positive events group and non-event group**.

	Non-event group	Positive event group	*p*
Sample	102	40	
Male, n (%)	73 (71.6)	25 (62.5)	0.396
Age, years (mean (SD))	46.32 (13.02)	56.15 (14.68)	<0.001*
BSA (mean (SD)) (m^2^)	1.76 (0.20)	1.66 (0.20)	0.008*
BMI (mean (SD)) (kg/m^2^)	23.88 (3.55)	24.49 (3.50)	0.359
Positive event, n (%)	0 (0.0)	40 (100.0)	<0.001*
Risk gene, n (%)	31 (30.40)	36 (90.0)	<0.001*
Obstruction, n (%)	21 (20.60)	18 (45.00)	0.006*
R_HR (mean (SD)) (bpm)	80.94 (12.58)	81.22 (14.25)	0.913
R_LVOT-PG (mean (SD)) (mmHg)	7.84 (2.19)	24.01 (13.98)	<0.001*
maxRW (mean (SD)) (mm)	19.74 (4.50)	22.38 (4.88)	0.003*
LVMI (mean (SD)) (g/m^2^)	145.12 (62.83)	147.86 (55.04)	0.801
R_WIS (mean (SD)) (m/s)	7.04 (3.66)	2.12 (1.26)	<0.001*
R_TTP (mean (SD)) (s)	4.70 (1.77)	6.17 (1.78)	<0.001*
R_PI (mean (SD)) (dB)	8.56 (3.31)	7.64 (3.70)	0.153
R_RT (mean (SD)) (s)	2.61 (1.75)	2.88 (1.89)	0.423
R_Sparse_num (mean (SD)) (number)	4.93 (3.76)	12.28 (5.02)	<0.001*
P_HR (mean (SD)) (bpm)	160.95 (18.55)	144.74 (26.31)	0.002*
P_LVOT-PG (mean (SD)) (mmHg)	21.16 (7.51)	38.44 (15.84)	0.007*
P_WIS (mean (SD)) (m/s)	10.44 (6.11)	2.57 (1.68)	<0.001*
P_TTP (mean (SD)) (s)	2.26 (1.24)	4.63 (2.02)	<0.001*
P_PI (mean (SD)) (dB)	10.29 (4.15)	7.16 (3.16)	<0.001*
P_RT (mean (SD)) (s)	0.97 (0.81)	2.47 (1.49)	<0.001*
P_Sparse_num (mean (SD)) (number)	4.37 (3.65)	12.32 (4.92)	<0.001*

*p < 0.05. BSA, body surface area; BMI, body mass index; LVMI, left ventricular mass index; HR, heart rate; maxRW, maximum thickness of the chamber wall; LVOT-PG, left ventricular outflow tract pressure gradient; Risk gene, pathogenic gene; R_WIS, the rest perfusion wash-in slope; P_WIS, the peak perfusion wash-in slope; R_TTP, rest perfusion time to peak; P_TTP, peak perfusion time to peak; R_PI, rest perfusion intensity; P_PI, peak perfusion intensity; R_RT, the rest and peak perfusion rise time; P_RT, peak perfusion rise time; R_ Sparse_num, the resting number of sparse myocardial segments for perfusion; P_Sparse_num, the peak number of sparse myocardial segments for perfusion.

### 3.7 Correlation Analysis

Positive events were moderately negatively correlated with resting WIS (r = –0.66), and peak WIS (r = –0.66). In contrast, moderate positive correlations were identified with peak TTP (r = 0.55). Positive events showed a weak correlation with the maxRW, as well as the resting and peak LVOT-PG (r = 0.26, r = 0.29, and r = 0.16).

### 3.8 The Predictive Performance of Stress Myocardial Perfusion for Positive Event

**Fig. 3.**
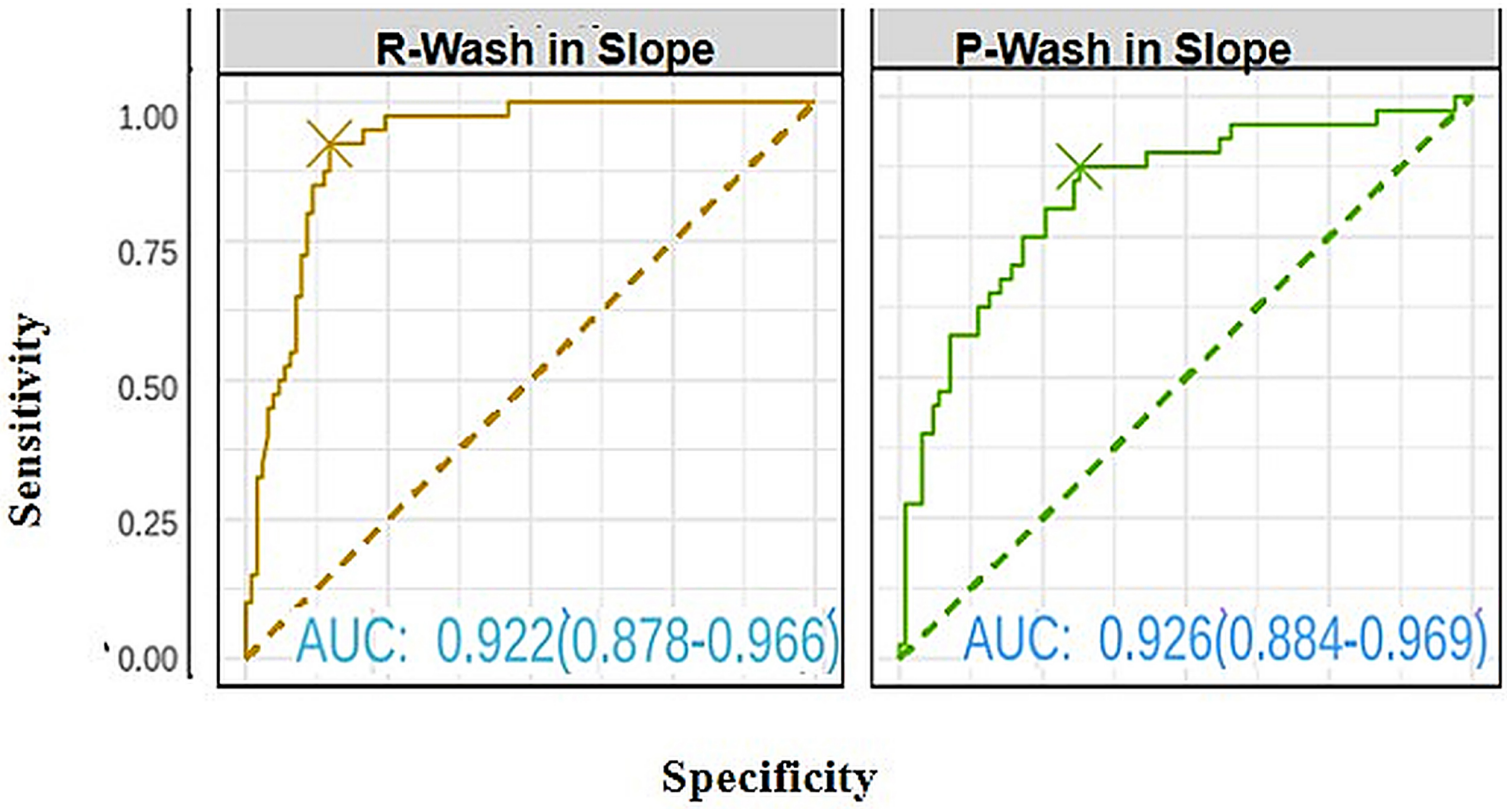
**ROC of P_WIS, R_WIS**. ROC, receiver operating characteristic; R_WIS, the rest perfusion wash-in slope; P_WIS, the peak perfusion wash-in slope.

As shown in Table 7 and Fig. 3, both resting and peak WIS had predictive performance for positive events. Given the exploratory nature of this study and the limited number of positive events (n = 40), multivariable adjustment was not performed. The ROC-derived predictive performance of resting and peak WIS supports their potential as candidate imaging markers for risk stratification in HCM, pending validation in larger cohorts.

**Table 7.** **Predictive efficacy of positive event**.

	Threshold	Specificity	Sensitivity	AUC	95% CI	DeLong test
P_WIS	5.64	79.41	97.50	0.93	0.88–0.96	NA
R_WIS	3.66	85.29	92.50	0.92	0.88–0.96	0.00
R_TTP	6.33	86.27	50.00	0.72	0.62–0.81	1.00
P_TTP	3.09	77.45	82.50	0.85	0.78–0.92	0.01
P_PI	10.07	53.92	85.00	0.73	0.63–0.81	0.00
R_Sparse_num	7.50	79.41	77.50	0.85	0.78–0.92	0.03
P_Sparse_num	7.50	84.31	77.50	0.88	0.81–0.94	0.00

AUC, area under the curve; R_WIS, the rest perfusion wash-in slope; P_WIS, the peak perfusion wash-in slope; R_TTP, rest perfusion time to peak; P_TTP, peak perfusion time to peak; P_PI, peak perfusion intensity; R_ Sparse_num, the resting number of sparse myocardial segments for perfusion; P_Sparse_num, the peak number of sparse myocardial segments for perfusion; NA, not applicable.

## 4. Discussion

Previous pathological and imaging studies have consistently demonstrated structural microvascular abnormalities in HCM, including intimal hyperplasia and luminal narrowing of intramural small arteries [17,18,19,20]. Stress cardiac magnetic resonance (CMR) with adenosine has further revealed perfusion defects in more than 40% of patients with HCM [21], indicating functional microcirculatory impairment. Li et al. [18] showed that exercise-induced myocardial perfusion reserve assessed by CMR predicts adverse outcomes in HCM patients. Komuro et al. [19] demonstrated through quantitative CMR that the time to peak was significantly correlated with ventricular tachycardia in HCM patients, indicating that this perfusion parameter may serve as a predictor of arrhythmic risk. Tran et al. [20] confirmed that microvascular dysfunction, assessed via transthoracic Doppler echocardiography, independently predicts adverse outcomes, even in non-obstructive phenotypes. Despite these advances, current methods have important limitations: some lack reliable quantitative perfusion measures, others rely on pharmacologic stress or ionizing radiation, and most have not been validated for long-term risk stratification in HCM. In contrast, our preliminary, exploratory study aims to address these gaps by employing exercise-stress MCE with quantitative analysis of WIS and perfusion intensity (PI) in a 5-year prospective cohort. We found that resting and peak WIS were significantly lower in patients who experienced adverse events, with complete loss of perfusion reserve. ROC analysis demonstrated that both resting and peak WIS had good predictive performance for adverse events (AUCs of 0.92 and 0.93, respectively). Thus, this preliminary study extends the current literature by providing a radiation-free, quantitatively robust exercise-based MCE approach to assess microvascular dysfunction and predict long-term outcomes in HCM, while acknowledging that these exploratory findings require validation in larger, multicenter cohorts.

In the positive event group, the rest and peak WIS and peak PI were significantly impaired with no increase from rest to peak exercise, suggesting a complete loss of reserve. In contrast, the non-event group showed significant increases in both parameters, indicating preserved reserve. Clinical characteristics analysis showed that 45% of patients with positive events had obstruction. To distinguish mechanical from intrinsic factors, subgroup comparisons were performed among non-obstructive, resting-obstructive, and latent-obstructive patient groups. Significant differences in resting and peak WIS were observed across subgroups. The resting obstructive subgroup had the lowest WIS values and showed complete loss of WIS reserve, whereas both non-obstructive and latent obstructive patients retained WIS reserve. No differences in PI or PI reserve were observed among subgroups. These findings suggest that sustained mechanical obstruction significantly affects WIS, consistent with Güçlü et al. [22], who reported that higher resting LVOT-PG is associated with lower myocardial capillary density. In the present study, 90% of patients in the positive event group carried risk genotypes (see Table 6). Comparisons between the VUS and genetic risk groups showed no significant difference in LVMI. However, resting and peak WIS and PI were significantly lower in the genetic risk group. Both groups retained WIS reserve, though the genetic risk group had worse reserve, whereas PI reserve was lost in that group. These findings suggest that genetic risk may affect PI and WIS independently of structural remodeling, consistent with previous studies. Kim et al. [23] found that the genotype-positive group had a higher incidence of adverse outcomes. Hughes et al. [24] demonstrated abnormal myocardial perfusion in carriers of sarcomeric gene mutations without left ventricular hypertrophy. Joy et al. [25] showed that patients with overt HCM exhibit reduced myocardial perfusion reserve under stress, and that these alterations are detectable in mutation carriers even in the absence of hypertrophy.

In addition to the subgroup analyses described above, the composition of the composite endpoint itself warrants discussion regarding its clinical heterogeneity. As shown in Table 1B, the composite endpoint in this study comprised five distinct clinical events with different frequencies: heart failure (37.5%), atrial fibrillation (22.5%), ICD implantation (15.0%), unexplained syncope (15.0%), and ventricular tachycardia (10.0%). These events differ substantially in their underlying pathophysiology and risk factors, which warrant careful consideration when interpreting the predictive value of WIS for the composite endpoint. Heart failure was the most common event in our cohort (15/40, 37.5%). In HCM, heart failure typically results from diastolic dysfunction, obstruction, or progression to systolic dysfunction [3]. Microvascular dysfunction has been linked to myocardial fibrosis and adverse remodeling, providing a plausible mechanistic link between reduced WIS and heart failure events [7]. Atrial fibrillation (AF) occurred in 9 patients (22.5%), consistent with the reported prevalence of approximately 20–25% in HCM populations [12]. AF in HCM is multifactorial, arising from both genetic factors and secondary hemodynamic changes [12,13]. Increased left atrial volume, along with impaired left atrial function, confers an increased likelihood of AF in HCM patients [11]. Importantly, the onset of AF is often accompanied by a decrease in functional status along with an increased risk of stroke and overall mortality [11]. A meta-analysis confirmed that AF is a risk factor for adverse survival outcomes in patients with HCM [10], and a large Korean cohort study (n = 8349) demonstrated that AF is independently associated with increased risks for stroke and all-cause mortality [11]. ICD implantation (6 patients, 15.0%) and ventricular tachycardia (VT) (4 patients, 10.0%) represent arrhythmic events directly linked to SCD risk. Current risk stratification for SCD in HCM relies on a combination of clinical factors, including maximal wall thickness, family history, unexplained syncope, and non-sustained ventricular tachycardia (NSVT) on Holter monitoring [3]. Microvascular ischemia is thought to contribute to an arrhythmogenic substrate by promoting fibrosis and electrical heterogeneity [7]. Unexplained syncope (6 patients, 15.0%) is a recognized risk marker for SCD in HCM, although its mechanisms are heterogeneous, including arrhythmic, hemodynamic (obstruction-mediated), and neurally mediated causes [3].

### Limitations

We must acknowledge several limitations of the present study. First, this is a single-center study with a relatively small sample size and only 40 endpoint events. Given the limited number of endpoint events and the single-center design, this study serves as a preliminary exploratory analysis aimed at identifying potential risk markers, rather than developing or validating a robust predictive model. Accordingly, to avoid overfitting and unstable statistical estimates, we chose not to perform multivariable logistic regression, time-to-event analyses (e.g., Cox regression), or detailed subgroup analyses stratified by event type (e.g., heart failure versus ventricular tachycardia). Internal validation techniques such as bootstrap resampling or cross-validation were not performed, which may affect the stability and generalizability of the AUC estimates. We acknowledge that unadjusted analyses cannot rule out confounding factors, including age and other covariates. Nevertheless, ROC analysis remains an appropriate initial step to quantify the univariate discriminative performance of candidate markers. The high AUC values (WIS) suggest that WIS carries substantial predictive signal. Collectively, these findings should be considered hypothesis-generating and require external validation in large-scale cohorts with comprehensive covariate adjustment and formal time-to-event analyses. Secondly, the composite endpoint included a variety of clinical events, including syncope, heart failure, ventricular tachycardia, atrial fibrillation, and ICD implantation. Given the limited number of events (n = 40), excluding the 9 cases of AF would reduce the sample size to 31, thereby compromising statistical power and increasing the risk of model overfitting. Because of the exploratory and hypothesis-generating nature of this study, we did not perform this sensitivity analysis at this stage; however, it may be addressed in future, larger-scale studies. Thirdly, no invasive assessment of microvascular function (such as coronary perfusion rate or endothelium-dependent response) was performed, limiting our ability to establish direct physiological correlations. Further studies are needed to address this issue in greater depth. Fourthly, although pathogenic genetic variation data were included, most genetic testing reports were sourced from different commercial institutions, resulting in insufficient sample size to support in-depth genotype-phenotype correlation analysis. Lastly, due to multiple factors, including differences in sociocultural backgrounds, family structures, economic conditions, and health literacy, a significant proportion of patients had missing baseline data (e.g., CMR examination, symptom-onset recall, family history of SCD, NSVT documentation, and prior echocardiography). This lack of longitudinal baseline data precluded direct comparison or validation of existing guideline-recommended risk models [3,9].

## 5. Conclusion

This preliminary, single-center, hypothesis-generating study suggests that WIS from exercise-stress MCE may be a useful marker for risk stratification in HCM, but larger, multicenter studies are needed to confirm these findings.

## Data Availability

The data that support the findings of this study are available from the first author upon reasonable request.
